# A novel negative regulatory mechanism of Smurf2 in BMP/Smad signaling in bone

**DOI:** 10.1038/s41413-020-00115-z

**Published:** 2020-11-23

**Authors:** Junichi Kushioka, Takashi Kaito, Rintaro Okada, Hiroyuki Ishiguro, Zeynep Bal, Joe Kodama, Ryota Chijimatsu, Melanie Pye, Masahiro Narimatsu, Jeffrey L. Wrana, Yasumichi Inoue, Hiroko Ninomiya, Shin Yamamoto, Takashi Saitou, Hideki Yoshikawa, Takeshi Imamura

**Affiliations:** 1 Department of Orthopaedic Surgery, Osaka University Graduate School of Medicine, 2-2 Yamadaoka, Suita, Osaka, 565-0871 Japan; 2 Bone and Cartilage Regenerative Medicine, The University of Tokyo, 7-3-1, Hongo, Bunkyo-ku, Tokyo, 113-8655 Japan; 3 Centre for Systems Biology, Lunenfeld-Tanenbaum Research Institute, Mount Sinai Hospital, 600 University Ave, Toronto, ON M5G 1X5 Canada; 4 Department of Cell Signaling, Graduate School of Pharmaceutical Sciences, Nagoya City University, 3-1 Tanabe-dori, Mizuho-ku, Nagoya, Aichi 467-8603 Japan; 5 Department of Molecular Medicine for Pathogenesis, Ehime University Graduate School of Medicine, 454 Shitsukawa, Toon, Ehime 791-0295 Japan; 6 Department of Gastroenterology and Metabology, Ehime University Graduate School of Medicine, 454 Shitsukawa, Toon, Ehime 791-0295 Japan; 7 Translational Research Center, Ehime University Hospital, 454 Shitsukawa, Toon, Ehime 791-0295 Japan

**Keywords:** Bone, Bone quality and biomechanics

## Abstract

Transforming growth factor-β (TGF-β) and bone morphogenetic protein (BMP) play important roles in bone metabolism. Smad ubiquitination regulatory factors (Smurfs) regulate TGF-β/BMP signaling via ubiquitination, resulting in degradation of signaling molecules to prevent excessive activation of TGF-β/BMP signaling. Though Smurf2 has been shown to negatively regulate TGF-β/Smad signaling, its involvement in BMP/Smad signaling in bone metabolism has not been thoroughly investigated. In the present study, we sought to evaluate the role of Smurf2 in BMP/Smad signaling in bone metabolism. Absorbable collagen sponges containing 3 μg of recombinant human BMP2 (rhBMP2) were implanted in the dorsal muscle pouches of wild type (WT) and *Smurf2*^*−/−*^ mice. The rhBMP2-induced ectopic bone in *Smurf2*^−^^/−^ mice showed greater bone mass, higher mineral apposition and bone formation rates, and greater osteoblast numbers than the ectopic bone in WT mice. In WT mice, the ectopic bone consisted of a thin discontinuous outer cortical shell and scant inner trabecular bone. In contrast, in *Smurf2*^*−/−*^ mice, the induced bone consisted of a thick, continuous outer cortical shell and abundant inner trabecular bone. Additionally, rhBMP2**-**stimulated bone marrow stromal cells (BMSCs) from *Smurf2*^*−/−*^ mice showed increased osteogenic differentiation. Smurf2 induced the ubiquitination of Smad1/5. BMP/Smad signaling was enhanced in *Smurf2*^*−/−*^ BMSCs stimulated with rhBMP2, and the inhibition of BMP/Smad signaling suppressed osteogenic differentiation of these BMSCs. These findings demonstrate that Smurf2 negatively regulates BMP/Smad signaling, thereby identifying a new regulatory mechanism in bone metabolism.

## Introduction

Transforming growth factor-β (TGF-β) and bone morphogenetic protein (BMP) play important roles in bone metabolism.^1–3^ Genetic mutations in TGF-β or BMP signaling pathway components cause heritable developmental bone diseases, and dysregulation of TGF-β or BMP signaling is often associated with osteoporosis or osteoarthritis.^2,4,5^ Through binding to membrane-localized serine/threonine kinase receptors, these ligands elicit specific signals that activate intercellular signal cascades, including those mediated by Smad proteins. Several Smad proteins play pivotal roles in this signaling; Smad2/3 mediate TGF-β signaling as TGF-β-specific receptor-regulated Smads (R-Smads), and Smad1/5/8 mediate BMP signaling as BMP-specific R-Smads. Additionally, Smad 6/7, which act as inhibitory Smads (I-Smads), are induced by TGF-β or BMP to suppress Smad signaling and establish a negative feedback loop. Extracellular antagonists, coreceptors in the cell membrane, phosphatase-induced dephosphorylation, and degradation/modification via the intracellular ubiquitin-proteasome system also regulate TGF-β and BMP signaling.^6,7^

Homologous to the E6-accessory protein C-terminus (HECT)-type E3 ubiquitin ligases, Smad ubiquitination regulatory factor (Smurf) 1 and Smurf2 regulate TGF-β/BMP signaling via ubiquitination, thereby leading to protein degradation to prevent excessive activation of TGF-β/BMP signaling.^8^ Smurf1 and Smurf2 share high sequence homology and have similar structural characteristics.^9^ *Smurf1*^*−/−*^; *Smurf2*^*−/−*^ (Smurf double knockout) mice display embryonic lethality.^10^ Smurf1 ubiquitinates Smad1/5 for proteasomal degradation and negatively regulates BMP/Smad signaling.^11,12^ *Smurf1*^*−/−*^ osteoblasts have exhibit increased osteoblastic differentiation, and *Smurf1*^*−/−*^ mice exhibit an increased bone mass phenotype.^10^ Additionally, Smurf2 ubiquitinates Smad2/3 for proteasomal degradation, thereby negatively regulating TGF-β/Smad signaling.^13,14^ *Smurf2*^*−/−*^ osteoblasts have also been shown to exhibit increased osteoblastic differentiation.^15^ In contrast to the increased bone mass phenotype of *Smurf1*^*−/−*^ mice, *Smurf2*^*−/−*^ mice exhibit a reduced bone mass and increased bone resorption.^15^ The enhanced interaction between Smad3 and vitamin D-induced receptor activator of nuclear factor-*κ*B ligand (RANKL) expression in osteoblasts from *Smurf2*^*−/−*^ mice and subsequent activation of osteoclasts have been reported to be among the mechanisms underlying these changes.^15^ However, many mechanisms underlying the regulation of bone metabolism by Smurfs remain unclear.

Though Smurf2 affects only TGF-β/Smad signaling, its involvement in BMP/Smad signaling in bone metabolism needs to be clarified. Therefore, we examined the involvement of Smurf2 in BMP/Smad signaling in a BMP-induced ectopic bone model, which reflects rapid enhancement of BMP signaling. By investigating intracellular signaling in vitro and in vivo in BMP2-induced ectopic bone of *Smurf2*^*−/−*^ mice, we evaluated the effects of Smurf2 on BMP/Smad signaling.

## Results

### *Smurf2*^*−/−*^ mice showed a small skeletal phenotype and increased osteoclastic bone resorption

**Fig. 1:**
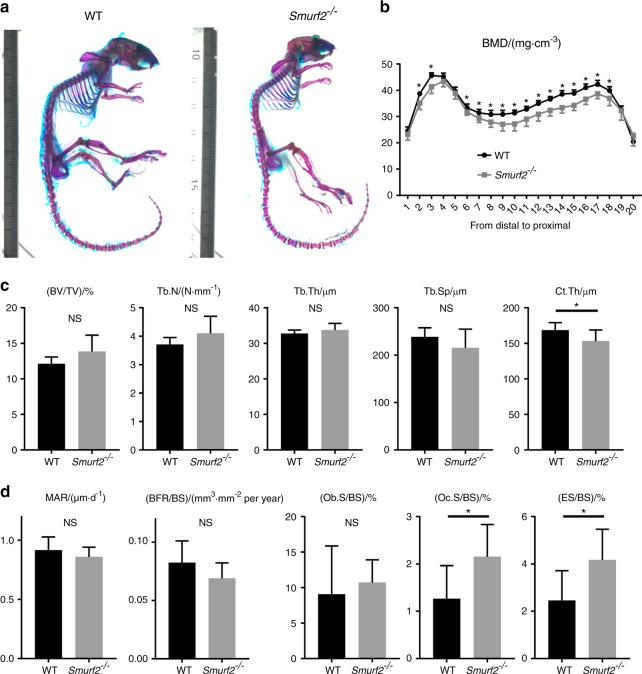
Skeletal evaluation. **a** Whole skeleton mouse littermates of either genotype. **b** BMD of femurs from 12-week-old mice of either genotype as determined by micro-CT analysis. The femurs were scanned in 20 longitudinal sections. The BMD of each section was measured from the distal to the proximal femur. **c** Micro-CT analysis of distal femurs from 12-week-old mice of either genotype. **d** Bone histomorphometric analysis of proximal tibias from 12-week-old mice of either genotype. (*n* = 8 per genotype; *P < 0.05; NS, not significant by Student’s *t* test)

The whole skeleton and femurs of *Smurf2*^*−/−*^ mice were investigated to evaluate the role of Smurf2 in bone metabolism homeostasis. In both overall size and weight, *Smurf2*^*−/−*^ mice were smaller than wild type (WT) control mice (Fig. 1a and Supplementary Fig. 1a–c). The distal femurs of *Smurf2*^*−/−*^ mice exhibited significantly lower bone mineral density (BMD) and cortical thickness (Ct.Th) than those of WT mice, whereas the distal femurs of *Smurf2*^*−/−*^ mice showed a normal bone volume fraction (BV/TV), trabecular thickness (Tb.Th), trabecular number (Tb.N), and trabecular spacing (Tb.Sp) (Fig. 1c).

The proximal tibias of *Smurf2*^*−/−*^ mice showed a normal BV/TV, Tb.Th, Tb.N, and Tb.Sp by histomorphometric bone analysis. In addition, analysis of the proximal tibias revealed no differences between *Smurf2*^*−/−*^ mice and WT mice with respect to the osteoid volume (OV/BV), osteoid surface (OS/BS), mineral apposition rate (MAR), and bone formation rate. However, the osteoclast surface and erosion surface in *Smurf2*^*−/−*^ mice was significantly increased, although the osteoblast surface was unchanged (Fig. 1d and Supplementary Fig. 1e).

These findings indicate that the loss of Smurf2 results in reduced skeletal size, reduced bone mass, and increased bone resorption.

### Recombinant human BMP2 (rhBMP2)-induced ectopic bone in *Smurf2*^*−/−*^ mice showed increased bone formation

**Fig. 2:**
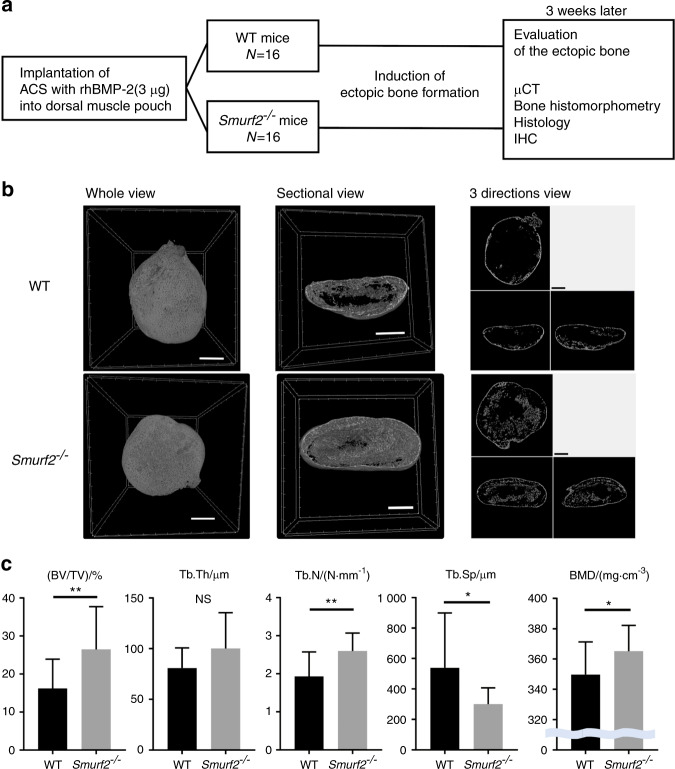
rhBMP2-induced ectopic bone formation model. **a** Schematic of the experimental design. **b** 3D micro-CT image of rhBMP2-induced ectopic bone (scale bar = 1 mm). **c** Micro-CT analysis of rhBMP2-induced ectopic bone. (*n* = 16 per genotype; ^*^P < 0.05; ^**^P < 0.01; NS, not significant by Student’s *t* test)

To examine the contribution of Smurf2 to BMP/Smad signaling in vivo, we next implanted rhBMP2-containing collagen sponges into *Smurf2*^*−/−*^ mice (Fig. 2a). In contrast to the (low bone mass) skeletal phenotype seen in *Smurf2*^*−/−*^ mice, the induced ectopic bone in *Smurf2*^*−/−*^ mice exhibited an increased bone mass with a higher BV/TV, Tb.N, and BMD and a lower Tb.Sp than observed in WT mice. In WT mice, the ectopic bone consisted of a thin outer cortical (Ct.) shell and scant inner trabecular (Tb.) bone. However, in *Smurf2*^*−/−*^ mice, the induced bone consisted of a thick outer Ct. shell and a full inner Tb. bone (Fig. 2b, c).

**Fig. 3:**
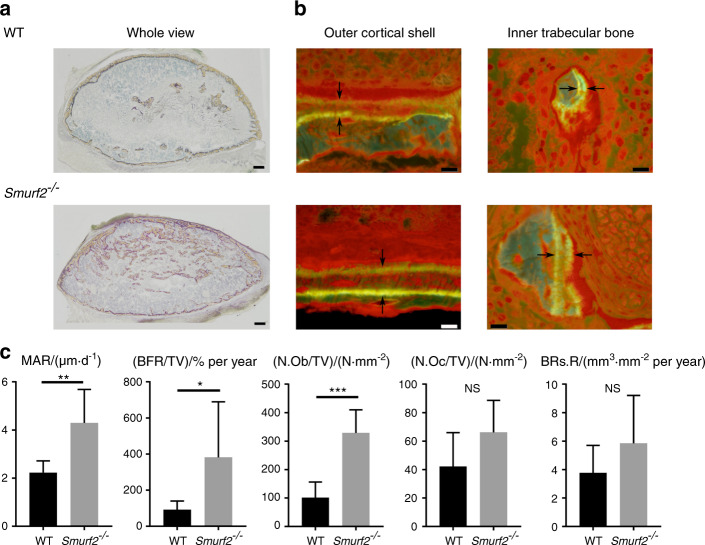
Bone histomorphometry of rhBMP2-induced ectopic bone. **a** Villanueva bone staining of the whole rhBMP2-induced ectopic bone (scale bar = 200 μm). **b** Fluorescence micrograph of the outer Ct. shell and inner Tb. bone in the rhBMP2-induced ectopic bone (scale bar = 10 μm). The arrows indicate bone labeled with tetracycline or calcein. **c** Bone histomorphometric analysis of rhBMP2-induced ectopic bone. (*n* = 6 WT mice, *n* = 7 *Smurf2*^*−/−*^ mice; *P < 0.05; **P < 0.01; ***P < 0.001; NS, not significant by Student’s *t* test)

Histomorphometric analysis of the induced ectopic bone was then conducted to enhance our understanding of the static and dynamic parameters of bone formation and resorption. The results of the static parameter analysis were consistent with those obtained by micro-computed tomography (micro-CT). Namely, the values of BV/TV, Tb.N, and osteoid bone parameters (OV/TV and OS/BS) of the ectopic bone were higher in *Smurf2*^*−/−*^ mice than in WT mice. Evaluation of the dynamic parameters of bone formation indicated that the MAR and bone formation rate were higher in *Smurf2*^*−/−*^ mice. In addition, the number of osteoblasts in the ectopic bone was higher in *Smurf2*^*−/−*^ mice. In contrast, the bone resorption rate, which is a dynamic parameter of bone resorption, and the number of osteoclasts were largely consistent between *Smurf2*^*−/−*^ and WT mice, although they were slightly increased in *Smurf2*^*−/−*^ mice (Fig. 3a–c and Supplementary Fig. 2).

**Fig. 4:**
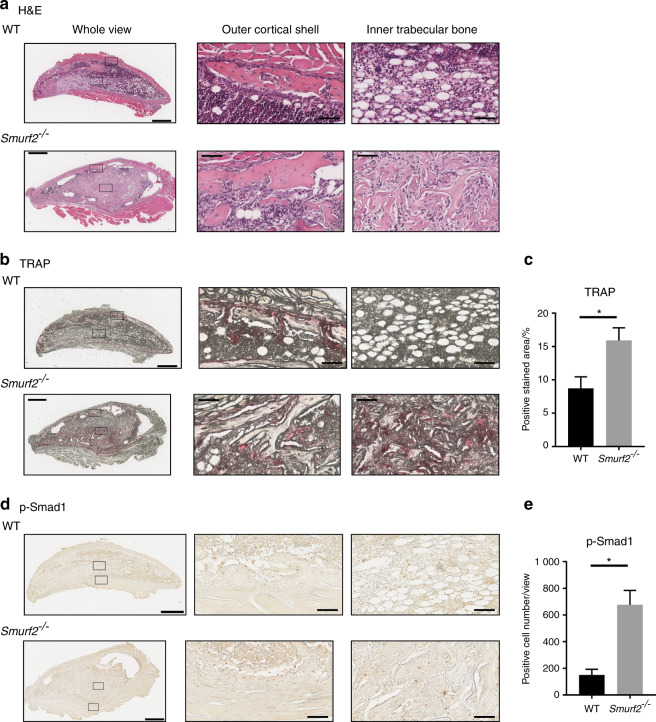
Histology of rhBMP2-induced ectopic bone. **a** H&E staining of rhBMP2-induced ectopic bone. **b** TRAP staining of rhBMP2-induced ectopic bone. **c** TRAP-stained area (*n* = 3; *P < 0.05 by Student’s *t* test). **d** Immunohistochemical staining of rhBMP2-induced ectopic bone with an anti-p-Smad1 antibody. **e** p-Smad1-positive cell numbers (*n* = 3; *P < 0.05 by Student’s *t* test). (Whole view, scale bar = 600 μm; outer Ct. bone and inner Tb. bone, scale bar = 60 μm)

Histological evaluation showed that the ectopic bone in *Smurf2*^*−/−*^ mice had less inner fat marrow and more Tb. bone than that in WT mice (Fig. 4a). The ectopic bone was well formed in the outer Ct. shell of both WT and *Smurf2*^*−/−*^ mice, and tartrate-resistant acid phosphatase (TRAP)-positive staining was observed in those areas. A TRAP-positive area was also observed in the inner Tb. bone of *Smurf2*^*−/−*^ mice, whereas the TRAP-positive area was very small in the inner Tb. bone of WT mice. Overall, the TRAP-positive area was larger in the *Smurf2*^*−/−*^ mice (Fig. 4b, c). Safranin O (SO) staining was not observed in mice of either genotype (Supplementary Fig. 3a). Immunohistochemistry revealed a larger number of phospho (p)-Smad1-positive cells in the ectopic bone of *Smurf2*^*−/−*^ mice (Fig. 4d, e), while only a limited number of p-Smad2-positive cells were observed in mice of either genotype (Supplementary Fig. 3b).

Thus, rhBMP2-induced ectopic bone in *Smurf2*^*−/−*^ mice exhibited a higher bone mass than the ectopic bone in WT mice. This increased mass was in contrast to the low bone mass phenotype of the skeletal bone of *Smurf2*^*−/−*^ mice. Additionally, immunohistological analysis showed evidence of Smad1 activation. These findings imply that Smurf2 is involved in the BMP/Smad signaling pathway.

### *Smurf2*^*−/−*^ BMSCs treated with rhBMP2 exhibited increased osteogenesis but not osteoclastogenesis in vitro

**Fig. 5:**
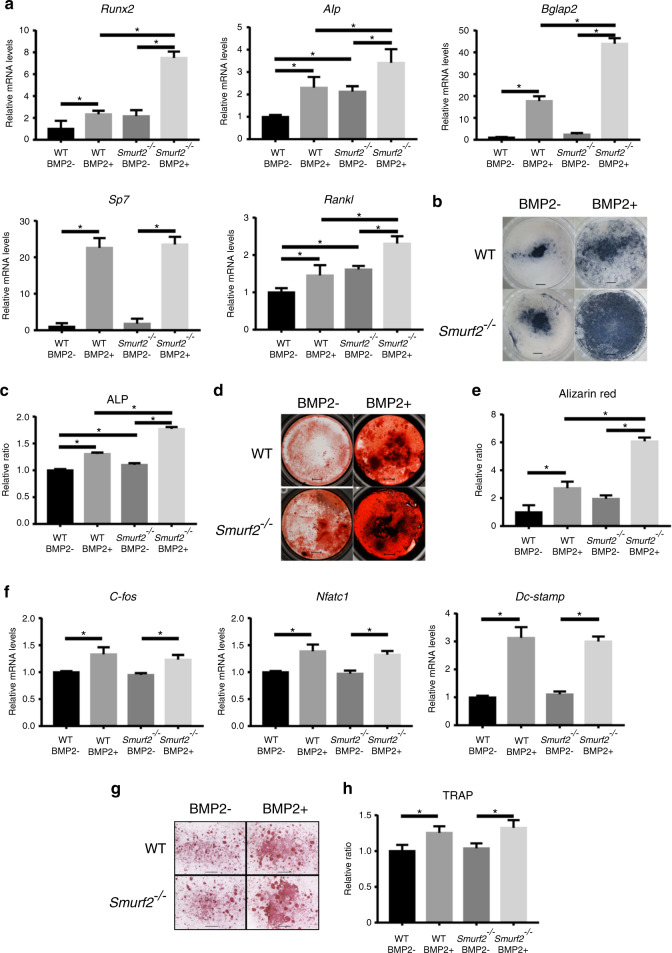
In vitro osteogenic and osteoclastic differentiation assays. **a** Quantitative real-time PCR analysis of BMSCs cultured in osteogenic medium with or without rhBMP2. **b**, **c** ALP staining of BMSCs cultured in osteogenic medium with or without rhBMP2 (scale bar = 2 mm). **d**, **e** Alizarin red staining of BMSCs cultured in osteogenic medium with or without rhBMP2 (scale bar = 2 mm). **f** Quantitative real-time PCR analysis of osteoclasts cultured in osteoclastogenic medium with or without rhBMP2. **g**, **h** TRAP staining of osteoclasts cultured in osteoclastogenic medium with or without rhBMP2. (scale bar = 1 mm). (*n* = 3 per group; *P < 0.05 by one-way ANOVA followed by the Bonferroni test)

To determine whether BMP/Smad signaling was enhanced by *Smurf2* deficiency, we performed in vitro osteogenic differentiation assays. Bone marrow stromal cells (BMSCs) isolated from mice of each genotype were cultured with or without rhBMP2 in osteogenic differentiation medium. Without rhBMP2 supplementation, BMSCs from *Smurf2*^*−/−*^ mice showed slightly higher expression of alkaline phosphatase (*Alp*) mRNA and higher levels of ALP staining than BMSCs from WT mice. Supplementation with rhBMP2 increased the expression of osteogenic genes (*Runx2*, *Alp, Bglap2*, and *Sp7*), ALP staining, and Alizarin red staining in BMSCs from mice of both genotypes. Among the groups, *Smurf2*^*−/−*^ BMSCs supplemented with rhBMP2 showed the greatest increase in the expression levels of osteogenic genes (*Runx2*, *Alp*, and *Bglap2*), ALP staining, and Alizarin red staining. Additionally, *Smurf2*^*−/−*^ BMSCs supplemented with rhBMP2 showed the highest expression level of *Rankl* (Fig. 5a–e).

We further performed an osteoclastic differentiation assay. Bone marrow cells isolated from mice of each genotype were cultured with or without rhBMP2 in osteoclastic differentiation medium. Supplementation with rhBMP2 increased the expression of osteoclastogenic genes (*c-fos*, *Nfatc1*, and *Dc-stamp*) and TRAP staining in osteoclasts from mice of both genotypes. However, osteoclasts from mice of each genotype cultured either with or without rhBMP2 showed similar expression levels of osteoclastogenic genes and TRAP staining (Fig. 5f–h).

These results suggest that BMSCs in *Smurf2*^*−/−*^ mice are more likely to undergo osteogenic differentiation than BMSCs in WT mice and that this tendency was further enhanced by rhBMP2 supplementation. These findings also imply that Smurf2 has a much lower effect on osteoclastogenesis than on osteogenesis.

### Smurf2 ubiquitinated Smad1/5, and BMP/Smad signaling was enhanced in mice with Smurf2 deficiency

Although Smurf2 has been reported to be involved exclusively in TGF-β/Smad signaling, our results suggest the involvement of Smurf2 in BMP/Smad signaling. To verify this involvement, we performed a ubiquitination assay and western blotting in order to confirm the effects of Smurf2 on BMP/Smad signaling in vitro.

**Fig. 6:**
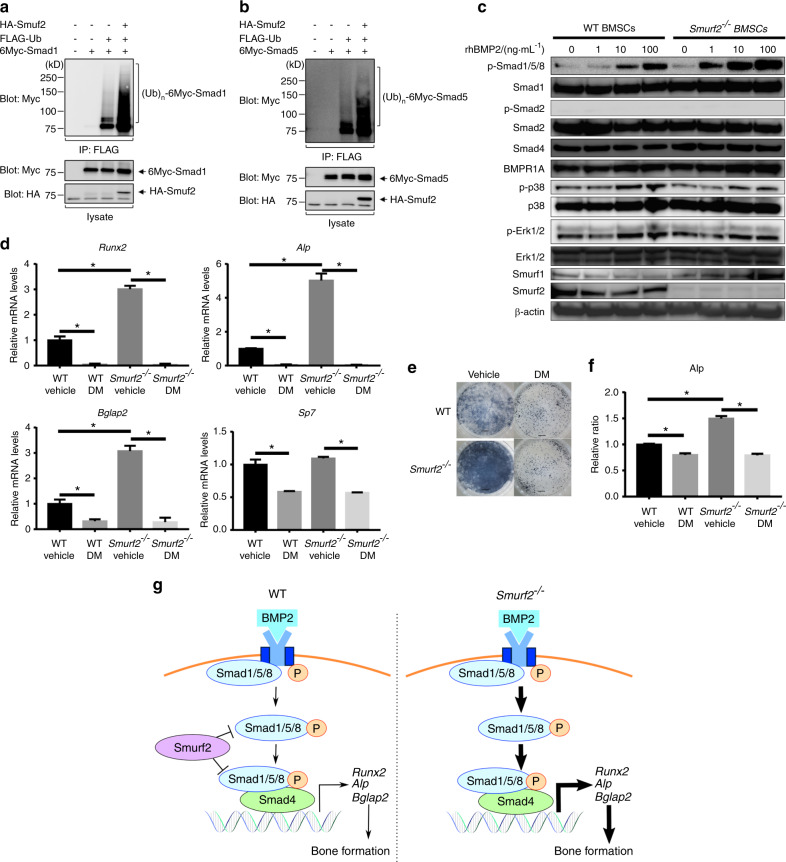
Effect of Smurf2 on BMP/Smad signaling. **a**, **b** Ubiquitination assay of Smad1 and Smad5 ubiquitination by Smurf2. **c** Western blot analysis with the indicated antibodies to evaluate expression levels in rhBMP2-stimulated BMSCs. **d** Quantitative real-time PCR analysis of BMSCs cultured in rhBMP2-supplemented osteogenic medium containing DMSO (Vehicle) or dorsomorphin (DM, 4 μmol·L^−1^). **e**, **f** ALP staining of BMSCs cultured in rhBMP2-supplemented osteogenic medium containing DMSO (Vehicle) or dorsomorphin (DM, 4 μmol·L^−1^) (scale bar = 2 mm). **g** Model of the BMP/Smad pathway in WT and *Smurf2*^*−/−*^ mice. (*n* = 3 mice per group; *P < 0.05 by one-way ANOVA followed by the Bonferroni test)

To determine whether Smurf2 can ubiquitinate Smad1 and Smad5, we evaluated the impact of Smurf2 on the ubiquitination of these Smads. As shown in Fig. 6a, b, Smurf2 induced the ubiquitination of Smad1 and Smad5.

After the addition of rhBMP2, BMSCs of *Smurf2*^*−/−*^ mice showed increased levels of p-Smad1/5/8 compared with those in BMSCs of WT mice. However, phosphorylated Smad2 was not observed in mice of either genotype. The BMSCs from mice of either genotype showed similar expression levels of Smad1, Smad2, Smad4, and BMPR1A. We further evaluated p38 and Erk1/2 to investigate non-Smad signaling pathways. BMSCs from mice of both genotypes showed increased levels of p-p38 and p-Erk1/2 after stimulation with rhBMP2. Thus, no difference in non-Smad signaling pathways was observed between the two genotypes. Increased expression of Smurf1 was observed only in rhBMP2-treated BMSCs from *Smurf2*^*−/−*^ mice (Fig. 6c and Supplementary Fig. 4). Additionally, with the addition of TGF-β3, BMSCs of *Smurf2*^*−/−*^ mice showed higher levels of p-Smad2 than WT BMSCs. Smurf1 expression was also slightly increased in BMSCs from *Smurf2*^*−/−*^ mice upon stimulation with TGF-β3 (Supplementary Fig. 5).

These findings indicate that Smurf2 exerts a suppressive regulatory effect on both BMP/Smad and TGF-β/Smad signaling.

### Inhibition of BMP/Smad signaling suppressed rhBMP2-induced osteogenic differentiation of *Smurf2*^*−/−*^ BMSCs

To further investigate the relationship between BMP/Smad signaling and Smurf2 in osteogenesis, we also performed a BMP/Smad signaling inhibition assay. Dorsomorphin was used to inhibit BMP-induced Smad1/5/8 phosphorylation.^16–18^ The levels of p-Smad1/5/8 were decreased in dorsomorphin-treated BMSCs from mice of both genotypes, but the expression of Smad1 did not change with dorsomorphin treatment (Supplementary Fig. 6). Thus, the inhibition of BMP/Smad signaling was confirmed.

Treatment with dorsomorphin suppressed the increase in the expression of osteogenic genes in *Smurf2*^*−/−*^ BMSCs to the same level as that in WT BMSCs. The enhanced ALP staining in *Smurf2*^*−/−*^ BMSCs was also decreased by dorsomorphin to the same level as that in WT BMSCs mice (Fig. 6d–f). Collectively, these results show that the BMP/Smad signaling pathway in *Smurf2*^*−/−*^ BMSCs plays a key role in the enhanced osteoblastic differentiation induced by rhBMP2.

## Discussion

This study revealed enhancement of in vivo bone formation, in vitro osteogenic differentiation, and increased BMP/Smad signaling with rhBMP2 supplementation in BMSCs from *Smurf2*^*−/−*^ mice. This study also demonstrated the direct ubiquitination of Smad1/5 by Smurf2 and showed decreased osteogenic differentiation via inhibition of BMP/Smad signaling in *Smurf2*^*−/−*^ mice. These findings imply that Smurf2 plays a novel suppressive role in BMP/Smad signaling by suppressing activated BMP/Smad signaling (Fig. 6g). TGF-β and BMP signaling exhibit crosstalk and are regulated by complex mechanisms. To investigate the effects of Smurf2 deficiency on BMP/Smad signaling in bone by a straightforward approach, we used a rhBMP2-induced ectopic bone formation model. In fact, activation of TGF-β/Smad signaling was not observed in rhBMP2-induced ectopic bone in *Smurf2*^*−/−*^ mice. Therefore, in this rhBMP2-induced ectopic bone formation model, BMP/Smad signaling was activated without causing a significant change in TGF-β/Smad signaling.

Although *Smurf2*^*−/−*^ mice have been reported to exhibit the formation of various tumors in old age,^19^ the involvement of Smurf2 in the skeletal phenotype of adult mice may be limited, because this phenotype is not severe in adult mice. The skeletal phenotype of the *Smurf2*^*−/−*^ mice used in this study was characterized by increased bone resorption and reduced bone mass, consistent with previous results.^15^ Based on the results of histological analyses, which showed that osteoblastic bone formation was consistent between WT and *Smurf2*^*−/−*^ mice and that osteoclastic bone resorption was increased in *Smurf2*^*−/−*^ mice, the increased function of osteoclasts is considered the primary cause of reduced bone mass. In contrast to the reduced bone mass phenotype observed in skeletal bone, the results in the rhBMP2-induced ectopic bone formation model, which reflects rapid bone formation, showed that ectopic new bone mass and osteoblastic bone formation were increased in *Smurf2*^*−/−*^ mice. These findings imply that the impact of Smurf2 deficiency differs between homeostatic bone metabolism in skeletal bone and the process of rapid ectopic bone formation.

The discrepancy between the skeletal bone phenotype and the results of the osteogenic differentiation assay in *Smurf2*^*−/−*^ mice might have been caused by differences in osteoblastic bone formation activity. The in vitro osteogenic differentiation assay revealed that the acute osteogenic differentiation process involving BMP/Smad signaling in *Smurf2*^*−/−*^ mice was enhanced by supplementation with rhBMP2. However, the in vivo skeletal bone phenotype in *Smurf2*^*−/−*^ mice represents a state of homeostasis in which a feedback or crosstalk mechanism can function to stabilize bone metabolism. Therefore, the conditions of the in vitro osteogenic differentiation assay were similar to those of rhBMP2-induced ectopic bone formation in *Smurf2*^*−/−*^ mice.

BMP has been reported to affect osteoclastogenesis indirectly and directly as well as exert osteoinductive effects on bone formation.^20,21^ In this study, rhBMP2-induced ectopic bone in *Smurf2*^*−/−*^ mice exhibited increases in the TRAP-positive area and number of osteoclasts. In vitro, *Smurf2*^*−/−*^ BMSCs showed enhanced *Rankl* expression when cultured in rhBMP2-supplemented medium. In contrast, in the osteoclastic differentiation assay, *Smurf2*^*−/−*^ osteoclasts showed a level of osteoclastogenesis similar to that of WT osteoclasts in rhBMP2-supplemented medium. A previous study also revealed no difference in osteoclastic differentiation between WT and *Smurf2*^*−/−*^ mice.^15^ These findings imply that increased RANKL expression in osteoblasts causes enhanced osteoclastic activity in *Smurf2*^*−/−*^ mice.

Smurf1 has been reported to cooperate with inhibitory Smads and suppress TGF-β superfamily signaling through several different mechanisms.^22,23^ Additionally, Smurf1 has been reported to regulate both BMP/Smad and TGF-β/Smad signaling.^24^ These reports, along with the results of the present study, imply that both Smurf1 and Smurf2 are involved in the regulation of both BMP/Smad and TGF-β/Smad signaling. In this study, western blot analysis revealed a minor increase in the expression of Smurf1 in *Smurf2*^*−/−*^ BMSCs stimulated with either rhBMP2 or TGF-β3. A previous study also reported increased expression of Smurf1 in Smurf2-deficient cells stimulated with TGF-β3.^25^ These results imply that Smurf1 and Smurf2 mutually interact to negatively regulate TGF-β/BMP signaling. A previous study reported that Smurf2 ubiquitinates and promotes the degradation of Smurf1.^26^ A possible reason for the increased Smurf1 expression observed in this study is that Smurf1 degradation is prevented under Smurf2-deficient conditions. Another possible explanation is the complementary function of Smurf1 under Smurf2-deficient conditions. Additional studies, however, are necessary to evaluate the interaction between Smurf1 and Smurf2.

This study had several limitations. First, rhBMP2-induced ectopic bone homeostasis differs from normal skeletal homeostasis. Whether BMP/Smad signaling is enhanced in the skeletal bone of *Smurf2*^*−/−*^ mice remains unclear. Further studies are needed to examine the interaction between BMP and Smurf2 in skeletal homeostasis. Second, we evaluated only mouse bone marrow-derived cells in vitro. However, similar to our data, another report indicated that *Smurf2* knockdown in human mesenchymal stem cells resulted in increased osteoblastic differentiation,^15^ suggesting that Smurf2 is also involved in osteogenic differentiation of mesenchymal cells. Among mesenchymal cells, platelet-derived growth factor receptor α-positive cells have been reported to be a major source of ectopic bone.^27,28^ Additional research is needed to clarify the role of Smurf2 in different cell types. Finally, although the ubiquitination assay revealed direct ubiquitination of Smad1 by Smurf2, the expression of Smad1 did not differ between WT and *Smurf2*^*−/−*^ BMSCs. In addition, recent studies indicated that the expression levels of Smads were not altered by Smurfs.^29–31^ The unchanged expression level of Smad1 despite the increased BMP/Smad signaling suggests that Smurf2 mainly influences the activation of BMP/Smad signaling. Additional research is necessary to determine the impact of Smurf2 in regulating the activation of BMP/Smad signaling.

In conclusion, this study revealed a novel negative regulatory mechanism of Smurf2 in BMP/Smad signaling in bone. These findings will help to improve the understanding of the intricate mechanisms controlling TGF-β/BMP signaling in bone.

## Materials and methods

### Mice

Previously described *Smurf2*^*−/−*^ mice^32^ were used in this study (Supplementary Fig. 7). These mice were backcrossed with C57BL/6J mice for more than 10 generations, and the mice were housed in a pathogen-free environment. The Animal Experimental Committee of Osaka University Graduate School of Medicine (No. 29-022-004) and Gene Modification Experiments Safety Committee of Osaka University (No. 04180) approved the animal studies.

### Skeletal staining

After sacrifice, 12-week-old mice were fixed overnight in 95% ethanol and were then transferred into acetone. The samples were then stained with Alizarin red and Alcian blue as described previously.^33^

### rhBMP2-induced ectopic bone formation model

The BMP2-induced ectopic bone formation model is a standard experimental system used to evaluate the osteogenic effect of BMP2 in mice.^34,35^ Absorbable collagen sponges (ACSs; CollaTape, Zimmer Dental, Carlsbad, CA, USA) were cut into a disc shape (5.5 mm in diameter and 1 mm thick) and were then soaked with 3 μg of rhBMP2 (R&D Systems, Minneapolis, MN, USA) dissolved in 10 μL of a sterile 4 mmol·L^−1^ HCl solution and freeze dried. The ACSs were implanted under the dorsal fascia of WT and *Smurf2*^*−/−*^ mice (*n* = 16 mice per group) under general anesthesia. Three weeks after this implantation, the induced ectopic bone was harvested and evaluated (Fig. 2a).

### Micro-CT analysis

Bones were scanned using high-resolution micro-CT (R_mCT, Rigaku, Tokyo, Japan) at 90 kV and 160 mA. Three-dimensional images of bones were analyzed using TRI/3D-BON software (RATOC System Engineering, Tokyo, Japan) and evaluated at a resolution of 20 μm per voxel. The relevant parameters of Tb. bone and Ct. bone were calculated as previously reported.^35–37^

### Bone histomorphometric analysis

Mice were subcutaneously injected with 20 mg·kg^−1^ tetracycline (Sigma-Aldrich, St. Louis, MO, USA) and 10 mg·kg^−1^ calcein (Sigma-Aldrich) at 3 days and 1 day prior to sacrifice, respectively, to label sites of active bone formation (*n* = 6 WT mice and *n* = 7 *Smurf2*^*−/−*^ mice). The ectopic bones were harvested, fixed with 70% ethanol, stained with Villanueva bone stain and subsequently embedded in methacrylate (Wako Pure Chemical Industries, Osaka, Japan) without decalcification. The resulting blocks were sliced into 5-μm-thick sections using a microtome (RM2255, Leica Biosystems, Wetzlar, Germany). Histomorphometric bone parameters were determined and subsequently expressed based on the standardized nomenclature for bone histomorphometry.^38^ Analysis was performed using semiautomatic image analysis software (System Supply, Nagano, Japan) under a fluorescence microscope (BX51, Olympus, Tokyo, Japan). An investigator who was blinded to the experimental groups measured the parameters.

### Histology and immunohistochemistry

Bones were fixed with 4% paraformaldehyde in PBS and decalcified with 20% EDTA. The bones were then dehydrated by incubation in an ethanol series and embedded in paraffin wax before being sliced into 3-μm-thick sections and stained with hematoxylin and eosin (H&E), SO/Fast Green, and toluidine blue according to standard protocols. In addition, TRAP staining was carried out based on standard protocols (Cosmo Bio, Tokyo, Japan).

Anti-p-Smad1 (1:100, ab73211) and anti-p-Smad2 (1:100, ab188334) antibodies were purchased from Abcam (Cambridge, UK), and an anti-Smurf2 (1:100, 12024) antibody was purchased from Cell Signaling Technology (Danvers, MA, USA). Information on antigen retrieval, protein blocking, primary antibodies, and reaction conditions is shown in Supplementary Table 1. Antibodies were detected with Histofine Simple Stain MAX PO (Nichirei Biosciences, Tokyo, Japan) and Simple Stain DAB Solution (Nichirei Biosciences). The area of TRAP staining was automatically measured with Aperio ImageScope software (Leica Biosystems), and the p-Smad1- and p-Smad2-positive cells were manually counted in 10 fields of view per pellet, as previously described.^39^

### In vitro osteogenic differentiation assay

Primary BMSCs were isolated from mouse femurs as described previously.^40^ In brief, after sacrifice, the hind limbs were aseptically removed from both WT and *Smurf2*^*−/−*^ mice, and soft tissues were removed. The femur bone marrow cavities were flushed with α-minimum essential medium (αMEM, Nacalai Tesque, Kyoto, Japan). The cells were cultured in αMEM supplemented with 10% fetal bovine serum (FBS; Sigma-Aldrich) and 1% antibiotic/antimycotic solution (A/A; Sigma-Aldrich). To assess the osteogenic differentiation ability of WT and *Smurf2*^*−/−*^ BMSCs induced by rhBMP2, these BMSCs were cultured in osteogenic medium (αMEM supplemented with 10 mmol·L^−1^ β-glycerol phosphate [β-GP, Calbiochem, San Diego, CA, USA], 50 μg·mL^−1^ ascorbic acid-2 phosphate [AA2P, Sigma-Aldrich], 10 nmol·L^−1^ dexamethasone [DXA, Sigma-Aldrich], 10% FBS, and 1% A/A) with or without 100 ng·mL^−1^ rhBMP2 (R&D Systems). Real-time PCR and ALP staining were performed on day 7, and Alizarin red staining was performed on day 21.

### In vitro osteoclastic differentiation assay

Primary bone marrow cells were isolated from mouse femurs as described above. The cells were cultured in αMEM supplemented with 10 ng·mL^−1^ macrophage colony-stimulating factor (MCS-F, R&D Systems), 10% FBS, and 1% A/A, as previously described.^41,42^ To examine the osteoclastic differentiation ability, WT and *Smurf2*^*−/−*^ osteoclasts were cultured in osteoclastogenic medium [αMEM supplemented with 10 ng·mL^−1^ MCS-F, 50 ng·mL^−1^ RANKL (R&D Systems), 10% FBS, and 1% A/A] with or without 100 ng·mL^−1^ rhBMP2 (R&D Systems). Real-time PCR and TRAP staining were performed on day 7.

### Real-time PCR assay

Cells were homogenized in TRIzol Reagent (Invitrogen, Carlsbad, CA, USA). Total RNA was extracted using a Direct-zol RNA kit (Zymo Research, Irvine, CA, USA) and was subsequently converted to cDNA using ReverTra Ace qPCR RT Master Mix (Toyobo, Osaka, Japan). Gene expression was measured via quantitative real-time PCR with SYBR Green Master Mix (Applied Biosystems, Foster City, CA, USA) in a Step One Plus Real-Time PCR System (Applied Biosystems). The primer sequences used for real-time PCR are listed in Supplementary Table 2. The mRNA levels were calculated from standard curves using the relative quantitation method and were then normalized to the GAPDH level for each sample.

### ALP staining

For ALP staining, cells were fixed and stained with ALP substrate solution [0.1 mg·mL^−1^ naphthol AS-MX (Sigma-Aldrich) and 0.6 mg·mL^−1^ fast violet B salt (Sigma-Aldrich) in 0.1 mol·L^−1^ Tris-HCl (pH 8.5)] for 30 min at 37 °C. Staining was then quantified with ImageJ software (National Institutes of Health, Bethesda, MD) as previously described.^43^

### Alizarin red staining

Cells were stained with Alizarin Red Solution (Muto Pure Chemicals, Tokyo, Japan) for 5 min at 20 °C. Alizarin red dye was extracted with 5% formic acid, and the absorbance at 415 nm was determined using a Multiskan GO instrument and SkanIt software (Thermo Fisher Scientific) as previously reported.^44^

### TRAP staining

Cells were fixed and stained with a TRAP staining kit (Cosmo Bio) for 60 min at 37 °C. Staining was quantified using ImageJ software (National Institutes of Health).

### Ubiquitination assay

The 6Myc-Smad1, 6Myc-Smad5, HA-Smurf1, HA-Smurf2, and Flag-ubiquitin (Ub) plasmids were described previously.^26,45^ COS7 cells were first transfected with the indicated plasmids using Lipofectamine 2000 (Invitrogen, Carlsbad, CA, USA). After 24 h, the cells were treated with 20 μmol·L^−1^ MG132 for 4 h and were then lysed in lysis buffer [20 mmol·L^−1^ Tris-HCl (pH 7.5), 120 mmol·L^−1^ NaCl, 1 mmol·L^−1^ EDTA, and 0.5% Triton X-100] supplemented with protease inhibitors. After 15 min of incubation on ice, the lysates were centrifuged at 15 000 r·min^−1^ and 4 °C for 5 min, and the supernatants were subjected to immunoprecipitation with anti-FLAG M2 agarose affinity gel (Sigma, St. Louis, MO, USA) at 4 °C for 2 h. After washing with lysis buffer (3×), the immunoprecipitated products were eluted on ice for 30 min with 3× FLAG peptide (Sigma) and resolved by SDS-PAGE.

### Western blotting

BMSCs from both WT and *Smurf2*^*−/−*^ mice were cultured for 24 h in serum-free αMEM. After serum starvation, the BMSCs were cultured with αMEM supplemented with different concentrations of rhBMP2 (0, 1, 10, and 100 ng·mL^−1^) or TGF-β3 (0 and 10 ng·mL^−1^; Pepro Tech, Inc., Rocky Hill, NJ, USA) for 30 min. The cells were lysed in RIPA buffer (Nacalai Tesque) containing a protease/phosphatase inhibitor cocktail (Cell Signaling Technology). Next, the protein concentrations in the cell lysates were quantified using a Pierce Rapid Gold BCA Protein Assay Kit (Thermo Fisher Scientific). Equal amounts of protein were separated on 4%–12% Bolt Bis-Tris Plus precast polyacrylamide gels (Invitrogen). The separated proteins were transferred onto a polyvinylidene fluoride membrane using a Mini Blot Module (Novex, San Diego, CA, USA). After blocking with PhosphoBLOCKER (Cell Biolabs, Inc., San Diego, CA, USA) for 1 h, the membrane was reacted overnight at 4 °C with a primary antibody and then for 1 h at room temperature with an anti-rabbit IgG horseradish peroxidase-conjugated antibody (7074, Cell Signaling Technology). Immunoreactive bands were detected with Amersham ECL Prime Western Blotting Detection Reagent (GE Healthcare, Little Chalfont, UK), and the membrane was visualized using an MF-ChemiBIS 3.2 imaging system (DNR Bio-Imaging Systems, Ltd., Israel). Information about the primary antibodies used for western blotting is presented in Supplementary Table 3. Band densities were quantified using ImageJ software (National Institutes of Health), and β-Actin was used as the loading control for internal normalization.

### In vitro BMP/Smad signaling inhibition assay

The BMP/Smad signaling inhibitor dorsomorphin (Abcam) was first dissolved in dimethyl sulfoxide (DMSO, Sigma) and then diluted in the medium described below (1:1 000). DMSO diluted in medium (1:1 000) was used as the vehicle. Similar to the procedure used for the osteogenic differentiation assay, BMSCs were cultured in rhBMP2-supplemented osteogenic medium (αMEM supplemented with 10 mmol·L^−1^ β-GP, 50 μg·mL^−1^ AA2P, 10 nmol·L^−1^ DXA, 100 ng·mL^−1^ rhBMP2, 10% FBS, and 1% A/A) containing DMSO (vehicle) or dorsomorphin (4 μmol·L^−1^). Real-time PCR and ALP staining were performed on day 7.

### Statistical analysis

All data are expressed as the mean ± standard deviation values. Data were analyzed using GraphPad Prism 7 (GraphPad Inc., La Jolla, CA, USA). Differences in the measured variables between two groups were analyzed using Student’s *t* test, while differences in measured variables among multiple groups were analyzed using one-way analysis of variance followed by the Bonferroni test. Differences with a *P* value of <0.05 were considered significant.

## Supplementary information

Supplementary Information
