# Supplementary material for: A novel negative regulatory mechanism of Smurf2 in BMP/Smad signaling in bone
Source: Bone Res. 2020 Nov 23;8:41. doi: 10.1038/s41413-020-00115-z (PMC7680794; doi:10.1038/s41413-020-00115-z)
Supplement: Supplementary file 1 — Supplementary Information [file 41413_2020_115_MOESM1_ESM.pdf]

Title:

A novel regulatory mechanism against BMP/Smad signaling by Smurf2 in bone

Junichi Kushioka, MD, PhD[a], Takashi Kaito, MD, PhD[a,*], Rintaro Okada, MD, PhD[a], Hiroyuki Ishiguro, MD, PhD[a], Zeynep Bal, MSc[a], Joe Kodama, MD[a], Ryota Chijimatsu, PhD[b], Melanie Pye, BSc[c], Masahiro Narimatsu, DDS, PhD[c], Jeffrey L. Wrana, PhD[c], Yasumichi Inoue, PhD[d], Hiroko Ninomiya, BSc.Agr[e], Shin Yamamoto, MD, PhD[f], Takashi Saitou, PhD[e,g], Hideki Yoshikawa, MD, PhD[a], Takeshi Imamura, MD, PhD[e,g,*]

[a] Department of Orthopaedic Surgery, Osaka University Graduate School of Medicine, 2-2 Yamadaoka, Suita, Osaka 565-0871, Japan

[b] Bone and Cartilage Regenerative Medicine, The University of Tokyo, 7-3-1, Hongo, Bunkyo-ku, Tokyo 113-8655, Japan

[c] Centre for Systems Biology, Lunenfeld-Tanenbaum Research Institute, Mount Sinai Hospital, 600 University Ave, Toronto, Ontario, M5G 1X5, Canada

[d] Department of Cell Signaling, Graduate School of Pharmaceutical Sciences, Nagoya City University, 3-1 Tanabe-dori, Mizuho-ku, Nagoya, Aichi 467-8603, Japan

[e] Department of Molecular Medicine for Pathogenesis, Ehime University Graduate School of Medicine, 454 Shitsukawa, Toon, Ehime 791-0295, Japan

[f] Department of Gastroenterology and Metabology, Ehime University Graduate School of Medicine, 454 Shitsukawa, Toon, Ehime 791-0295, Japan

[g] Translational Research Center, Ehime University Hospital, 454 Shitsukawa, Toon, Ehime 791-0295, Japan

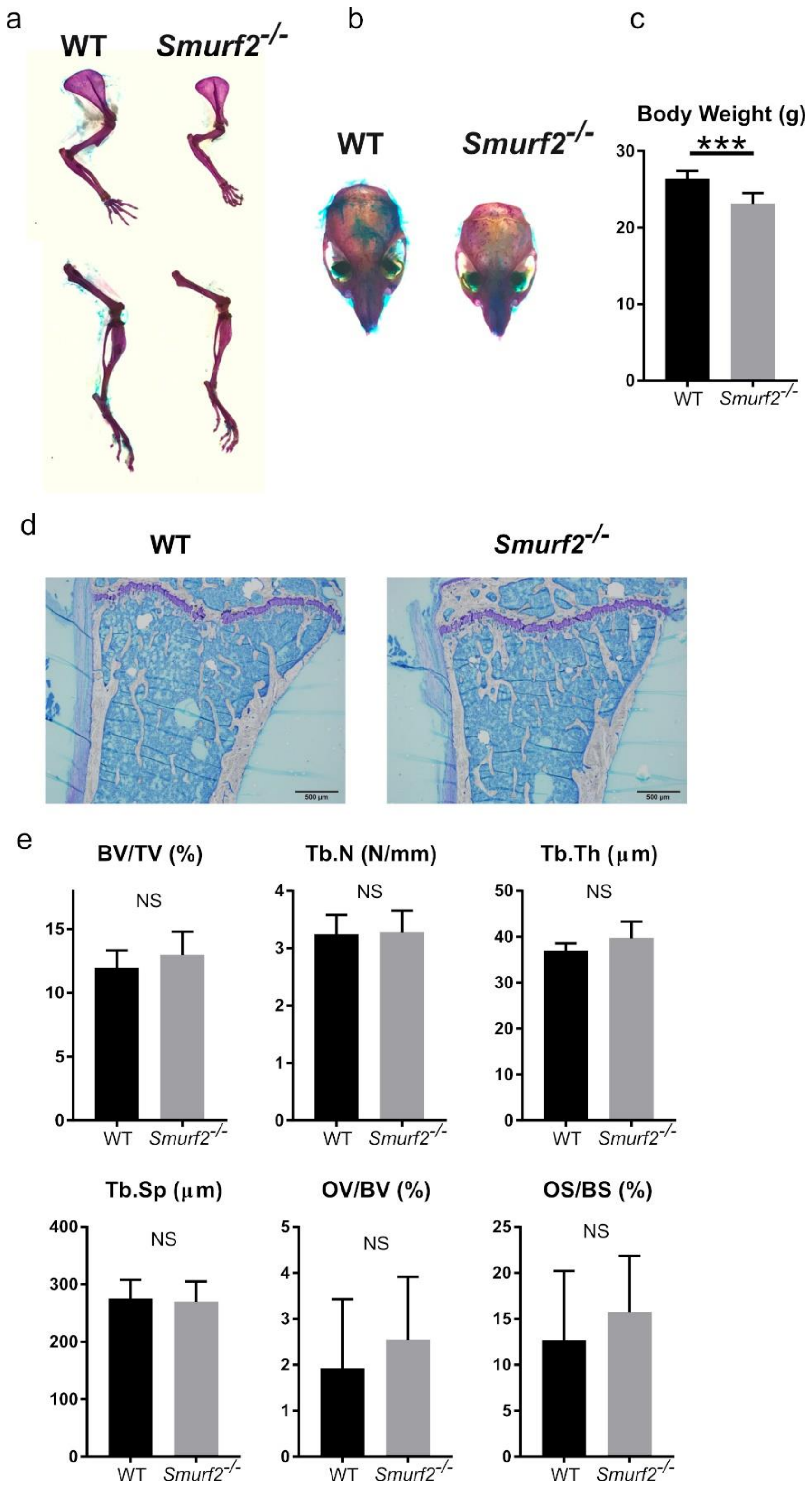

Supplementary Figure 1. Skeleton evaluation

(a) Alcian blue/Alizarin red staining of forelimb and hindlimb from 12-week-old WT and

Smurf2$^{-/-}$ mice littermates.

(b) Alcian blue/Alizarin red staining of the head of 12-week-old WT and *Smurf2*$^{-/-}$ mice.

(c) Bodyweight of 12-week-old WT and *Smurf2*$^{-/-}$ mice.

(d) Toluidine blue staining of the proximal tibia from 12-week-old WT and Smurf2-/- mice (scale bar=500 μm).

(e) Bone histomorphometric analysis of proximal tibia from 12-week-old WT and Smurf2-/- mice.

(WT; wild type, BV/TV; bone volume fraction, Tb.N; trabecular number, Tb.Th; trabecular thickness, Tb.Sp; trabecular separation, OV/BV; osteoid bone volume fraction, OS/BS; osteoid bone volume per bone surface,). Data represent mean ± S.D. (n=8 for each genotype, *; $p<0.05$, ***; $p<0.001$, NS; not significant by Student's t-test).

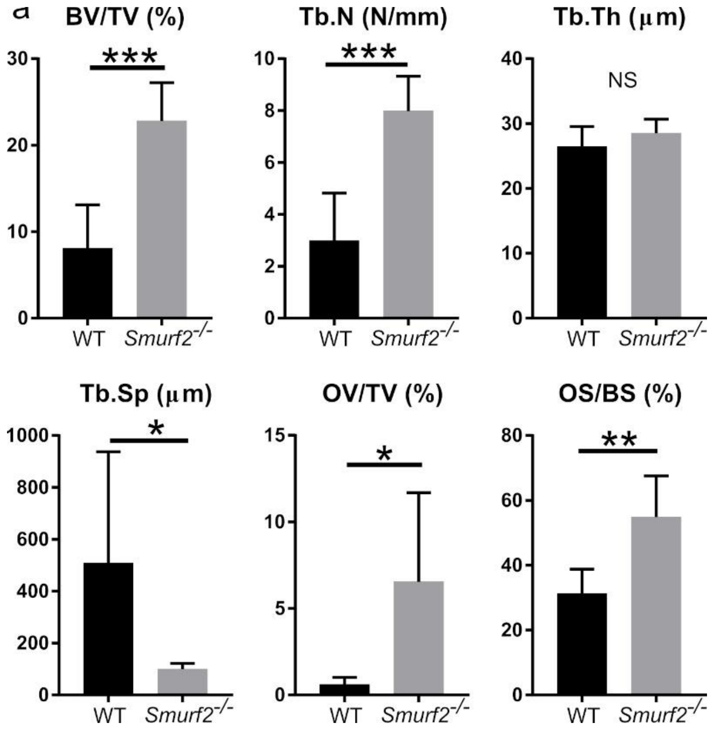

Supplementary Figure 2. Bone histomorphometry of the rhBMP2-induced ectopic bone

(a) Bone histomorphometric analysis of rhBMP2-induced ectopic bone.

(rhBMP2; recombinant human bone morphogenetic protein 2, WT; wild type, BV/TV; bone volume fraction, Tb.N; trabecular number, Tb.Th; trabecular thickness, Tb.Sp; trabecular separation, OV/TV; osteoid bone volume fraction, OS/BS; osteoid bone volume per bone surface). Data represent mean ± S.D. (n=6 for WT mice, n=7 for Smurf2-/- mice, *; $p<0.05$, **; $p<0.01$, ***; $p<0.001$, NS; not significant by Student's t-test).

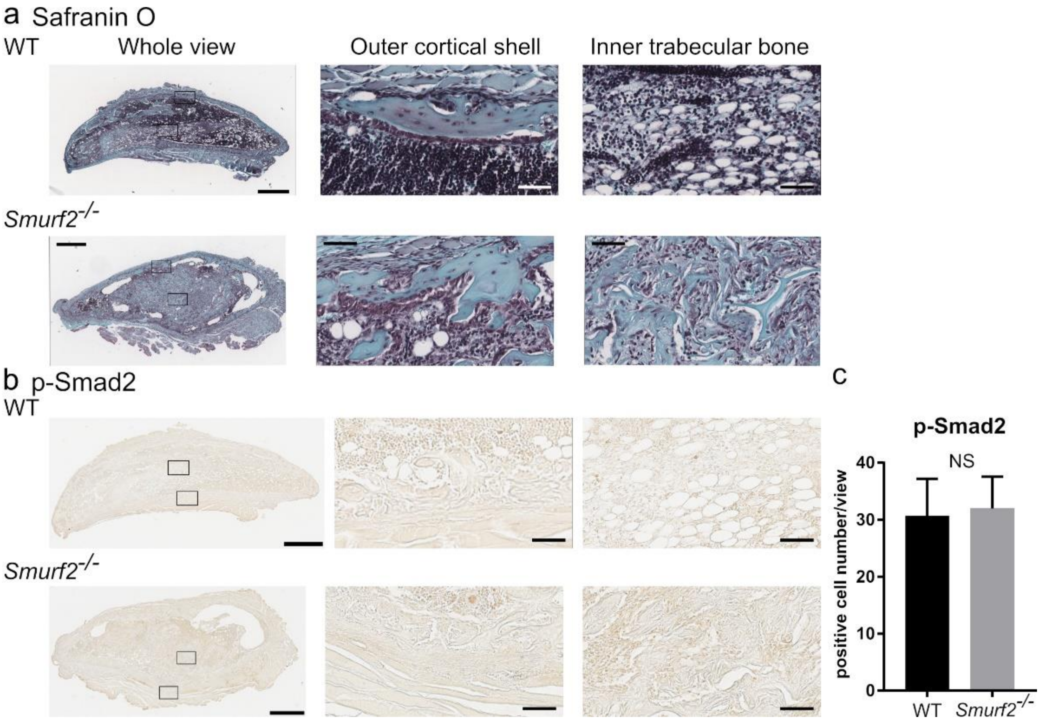

Supplementary Figure 3. Histology of the rhBMP2-induced ectopic bone

(a) Safranin O staining of the rhBMP2-induced ectopic bone.

(b) Immunohistochemistry stained with anti-p-Smad2 of the rhBMP2-induced ectopic bones.

(c) p-Smad2 positive cell numbers (n=3, NS; not significant by Student's t-test)

(WT; wild type, rhBMP2; recombinant human bone morphogenetic protein 2, Whole view; scale bar=600 μm, Cortical bone and Trabecular bone; scale bar=60 μm).

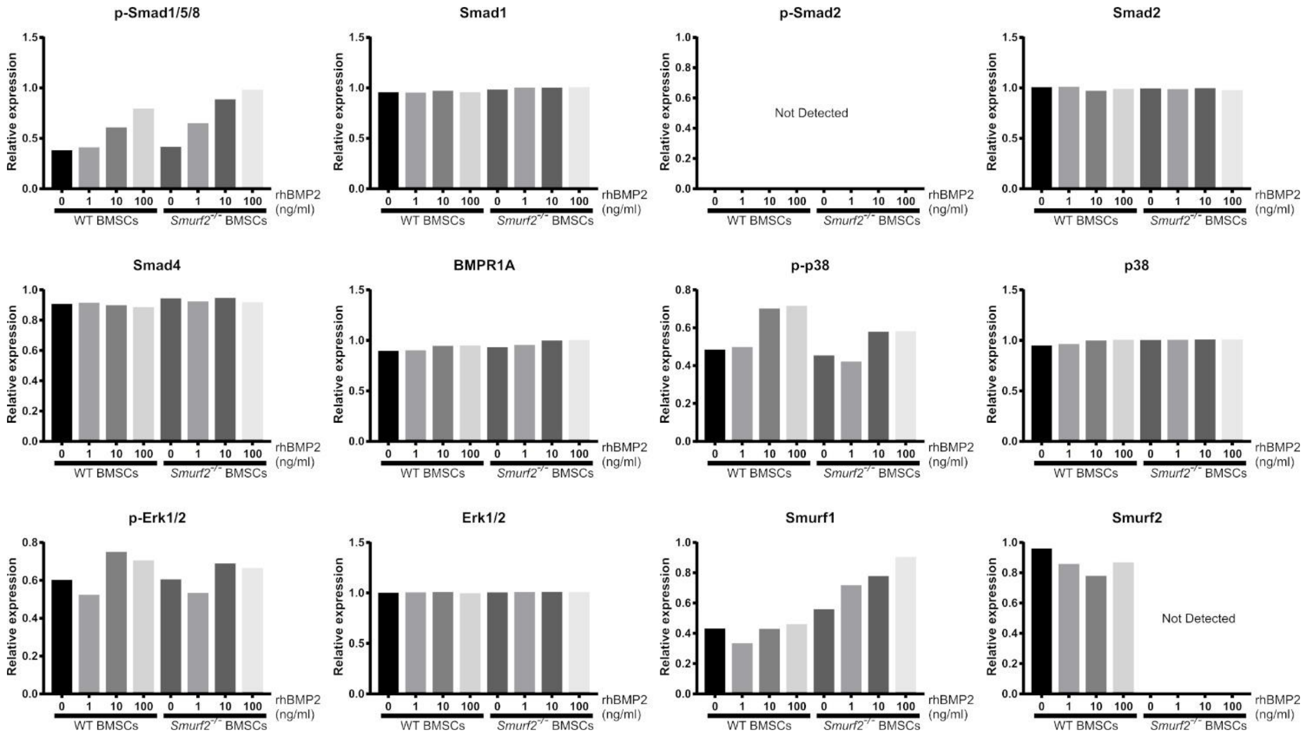

Supplementary Figure 4. Quantitative western blotting of BMSCs stimulated by rhBMP2

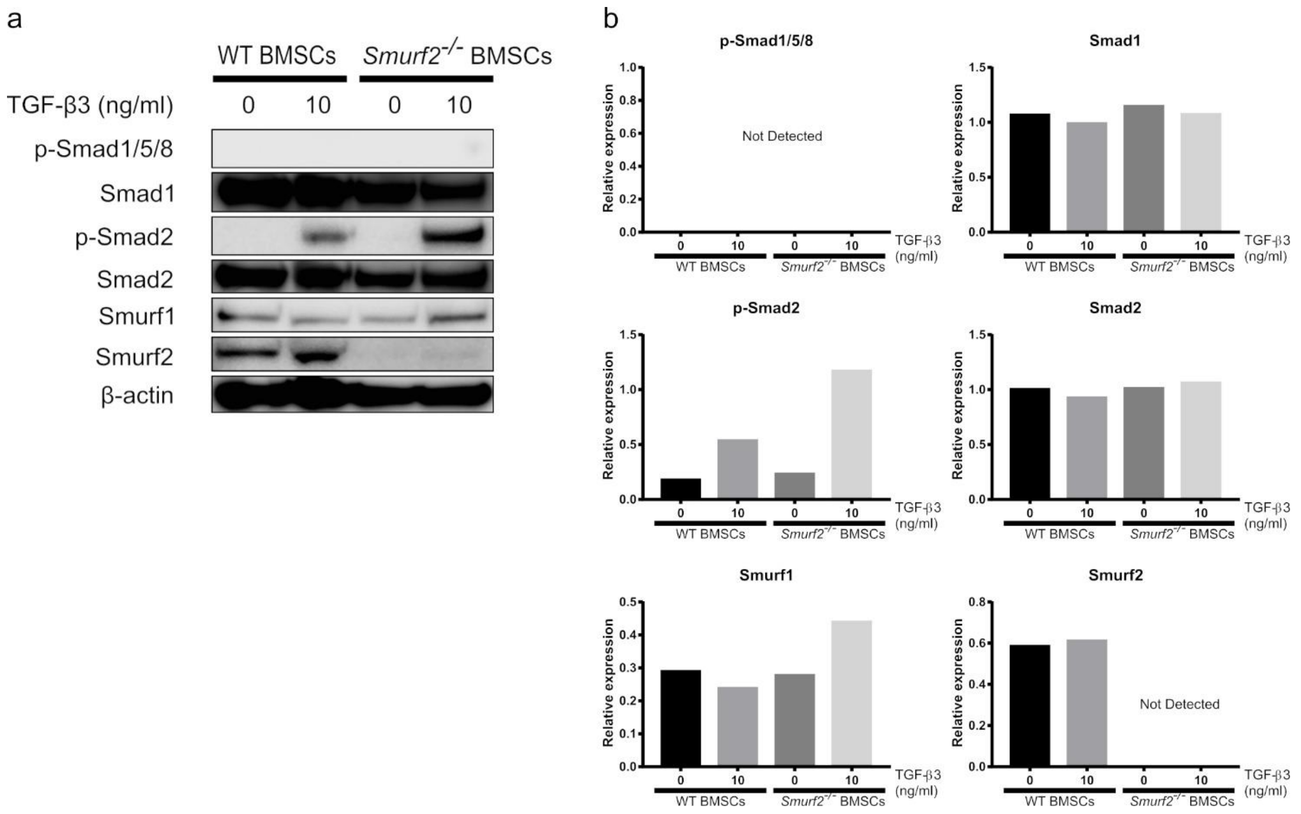

Supplementary Figure 5. Western blotting of BMSCs stimulated by TGF-β3

(a) Western blotting; Expression of the indicated antibodies of the BMSCs stimulated by TGF-β3.

(b) Quantitative western blotting of BMSCs stimulated by TGF-β3.

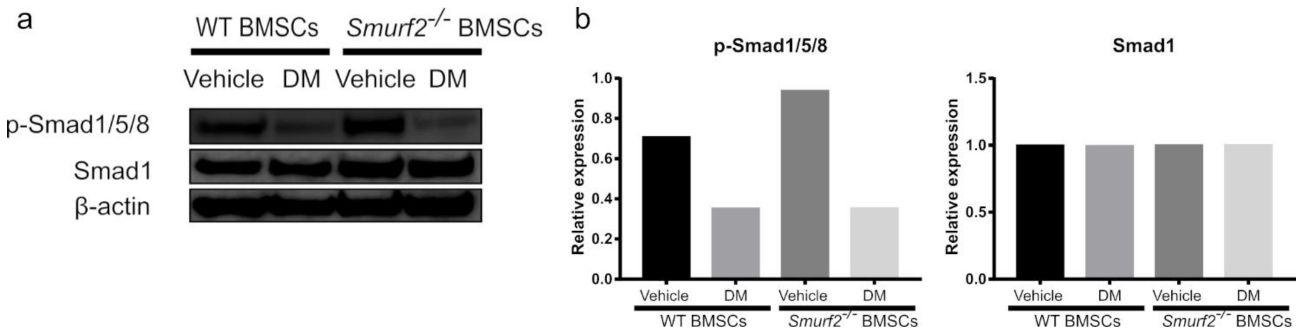

Supplementary Figure 6. Western blotting of BMSCs stimulated by vehicle or dorsomorphin

(a) Western blotting; Expression of the indicated antibodies of the BMSCs stimulated by vehicle or dorsomorphin (DM).

(b) The numerical results of the western blotting of BMSCs stimulated by vehicle or dorsomorphin (DM).

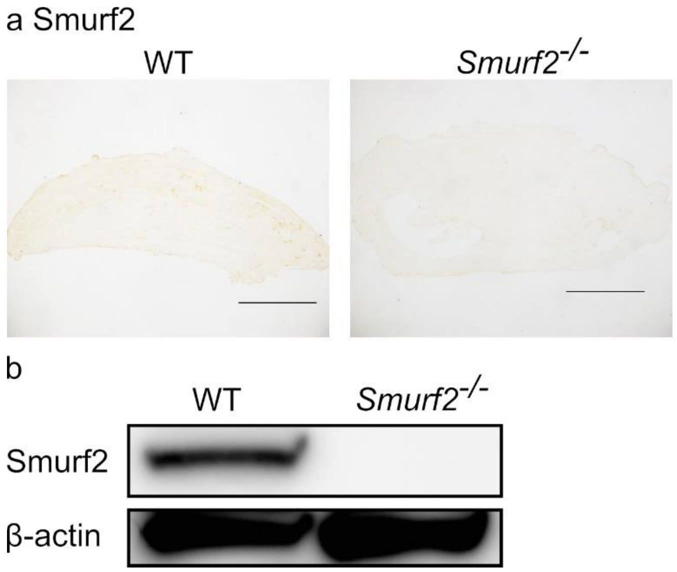

Supplementary Figure 7. Confirmation of Smurf2 deficiency.

(a) Immunohistochemistry stained with anti-Smurf2 of the rhBMP2-induced ectopic bones (scale bar=1000 μm).

(b) Western blot assay; Expression of Smurf2 and β-actin of the rhBMP2-induced ectopic bone.

(WT; wild type, rhBMP2; recombinant human bone morphogenetic protein 2)

Supplementary Table 1. Immunostaining details

Target Antigen	Company	Catalog No.	Antigen Retrieval	Dilution	Reaction
Phospho-Smad1	Abcam (Cambridge, UK)	73211	Antigen retrieval buffer (Abcam), 70°C, 60 min	1/100	20°C, 1 hr
Phospho-Smad2	Abcam	188334	Antigen retrieval buffer (Abcam), 70°C, 60 min	1/100	20°C, 1 hr
Smurf2	Cell Signaling Technology (Danvers, MA)	12024	Antigen retrieval buffer (Abcam), 70°C, 60 min	1/100	20°C, 1 hr

Supplemental Table 2. Primer sequences for gene expression analysis

Gene	Forward	Reverse
Gapdh	GGGTGTGAACCACGAGAAAT	ACTGTGGTCATGAGCCCTTC
Runx2	AGGCACAAAGAAGCCATAC	AATGAGTGAGGGAAGGGT
Bglap2	TGGCGACACTTACCGAGCTT	CCATGCCCCTTGTAGTAGCTGTA
Sp7	CATCTGCCTGACTCCTTGGGAC	GCTGAAAGGTCAGCGTATGGC
Alp	CCCAAGGAAAAGAAGCACGTC	ACATTAGGCGCAGGAAGGTCA
Rankl	CAGCATCGCTCTGTTCCTGTA	CTGCGTTTTCATGGAGTCTCA
C-fos	AAACCGCATGGAGTGTGTTGTTCC	TCAGACCACCTCGACAATGCATGA
Nfatc1	CCGTTGCTTCCAGAAAATAACA	TGTGGGATGTGAACTCGGAA
Dc-stamp	GACCTTGGGCACCAGTATTT	CAAAGCAACAGACTCCCAAA

Supplemental Table 3. Antibodies used in western blotting

Target Antigen	Company	Catalog No.	Dilution
Phospho- Smad1/5/8	Cell Signaling Technology (Danvers, MA)	13820	1/1000
Smad1	Cell Signaling Technology	6944	1/1000
Phospho-Smad2	Cell Signaling Technology	3108	1/1000
Smad2	Cell Signaling Technology	5339	1/1000
Smad4	Cell Signaling Technology	38454	1/1000
BMPR1A	Abcam	174815	1/1000
Phospho-p38	Cell Signaling Technology	9215	1/1000
p38	Cell Signaling Technology	9212	1/1000
Phospho-Erk1/2	Cell Signaling Technology	9101	1/1000
Erk1/2	Cell Signaling Technology	4695	1/1000
Smurf1	Proteintech, Inc. (Chicago, IL)	55175	1/1000
Smurf2	Cell Signaling Technology	12024	1/1000
β-actin	Cell Signaling Technology	4970	1/1000
